# Supplementary material for: Severe outcomes of malaria in children under time-varying exposure
Source: Nat Commun. 2024 May 14;15:4069. doi: 10.1038/s41467-024-48191-7 (PMC11094066; doi:10.1038/s41467-024-48191-7)
Supplement: Supplementary file 1 — Supplementary Information [file 41467_2024_48191_MOESM1_ESM.pdf]

Supplementary Text

Supplementary Notes

Supplementary Note 1. *Details of the contemporary and historical datasets.*
Supplementary Note 2. *Detailed description of OpenMalaria model.*
Supplementary Note 3. *Details on regression models, model selection criteria, computation of prediction intervals and evaluation of predictions.*
Supplementary Note 4. *Evaluation of the major epidemiological determinants in the scenario modeling framework.*
Supplementary Note 5. *Recovery of hospitalization risk by age group*

Figures S1 to S10

Tables S1 to S4

References 1 to 46

Supplementary Text

Supplementary Note 1. *Details of the contemporary and historical datasets.*

Information of the datasets has been previously published elsewhere [1–3].

Contemporary data

The dataset comprised information from 35 time-sites, and included Apac A (2017-18), Apac B (2017-18), Bungoma A (2019-20), Bungoma B (2019-20), Busia (2015-16 and 2017-20, ,Homa Bay (2019-20), Jinja A (2017-18), Jinja B (2012-13 and 2017-18), Kilifi A (2007-09, 2010-15 and 2017-19), Kilifi B (2007-09, 2010-15, 2017-19), Kabale A (2017-18), Kabale B (2017-18), Kakamega (2015-16 and 2017-18), Mubende A (2017-18), Mubende B (2017-18), Muheza A (2006-07), Muheza B (2006-07), Muheza C (2006-07), Siaya A (2010-13), Siaya B (2010-13), Siaya C (2010-13), Tororo A (2012-13 and 2017-19), Tororo B (2012-13 and 2017-19), Tororo C (2012-13), and Vihiga (2017-18). See figure S2 supplementary material in Paton et al.[3] showing all catchment populations and their distance to the hospital where inpatient records were collected.

Malaria inpatient admissions. Malaria admissions were defined based on clinicians' reviews on all available clinical, laboratory and radiological information. Children aged less than 3 months were excluded for simplicity as the influence of maternal immunity complicates the analysis, and severe malaria incidence is very low below 3 months. Children with underlying conditions that may have precipitated admission were excluded, including sickle cell disease, HIV, tuberculosis, malignancies, trauma, epilepsy, poisoning, snake/animal bites and measles. Children with other coincidental secondary diagnoses were retained, accepting that comorbidity was common, or hard to define as primary, secondary or co-primary.

Catchment populations. Residential data for each admission was matched to the smallest possible area, defined using national census, located within 30 km of the hospital but excluding urban areas. The definition of the catchment population aimed to avoid competition with other facilities, allow computation from available census-data granularity, standardize across sites within the contemporary dataset and allow comparability with the historical dataset, and reduce the case underascertainment within the "whole" hospital catchment population under the assumption that larger distances implied roughly more missing of cases. Population counts among the selected catchments were derived from the most contemporary national census and projected forwards or backwards using district level intercensal growth rates. Age-structures of each population were corrected to single year age groups 3-11 months to 9 years by applying rural household age structures provided for the nearest time-regional matched demographic household survey data. Age specific person years of observation were

adjusted for the months of observation included in each temporal series. The exceptions were three sites in Kilifi (Kenya) where actual household continuous population surveillance data were used to define age specific person years of observation. Catchments were selected to avoid competition with other facilities offering admission.

Community parasite prevalence. Each hospital catchment population was paired with a community parasite prevalence estimate derived from empirical surveys. Spatially and temporally disaggregated community-based household parasite prevalence survey data were used at six time-site periods in Kenya (Kilifi and Siaya) and four time-site periods in Uganda (Tororo and Jinja 2012-2013). Surveys of malaria parasite prevalence among school children were established during or within 4 months of the pediatric ward surveillance in western Kenya 2014-2019 (Busia, Bungoma, Homa Bay, Kakamega and Vihiga) and Uganda 2017-2019 (Tororo, Apac, Mubende, Kabale and Jinja). Published estimates of parasite prevalence at three sites in Muheza, Tanzania were extracted from literature. To standardize parasite prevalence across sites, we implemented a version of the conversion algorithm by Smith et al. (2007) [4] which standardizes parasite surveys to the 2-10 years age range.

Historical dataset

Similar criteria were used to collate both hospitalization datasets explicitly to allow comparison. Thus, comparable approaches to those used in the contemporary dataset were used to estimate malaria hospitalization incidence -with malaria as primary reason of admission- among children aged 1 month to 9 years at 6 time-site locations including Bakau, The Gambia (1991-94), Snow et al. (1997) [1]; Kilifi Township, Kenya (1993-96), Robert Snow unpublished data; Foni Kansala, The Gambia (1994-95), Geisler Schnieder and Robert Snow, unpublished data; Sukuta, The Gambia; Kilifi North Kenya (1990-95: Snow et al., (1997) [1]; and Mponda, Malawi (1994-95), Slutsker et al. (1994) [5]. Again, similar criteria were used for collating catchment population based on census and census projections, matched to an area within 15 km of essential clinical services, as well as the community prevalence based on cross-sectional parasitological surveys among children 3 months to 10 years.

Supplementary Note 2. *Detailed description of OpenMalaria model of malaria transmission dynamics*

Overview: Individual-based model of malaria transmission

For our model simulations and scenario analysis we used our individual-based model of *Plasmodium falciparum* malaria transmission and disease dynamics, OpenMalaria previously described and calibrated elsewhere [6–8]. OpenMalaria is documented in detail in our online source code and wiki (https://github.com/SwissTPH/openmalaria/wiki) and has been validated against several field studies, used in many studies on the epidemiological effects of various intervention, as well as compared to existing models [6,9–11]. OpenMalaria currently comprises 14 model variants, with each model variant with distinct assumptions of its epidemiology and transmission components (14). For our current study, the "base" simulation model was used, first detailed in [12], and again in [7]. A single-layer Bayesian optimization approach has been implemented to solve the multidimensional, multi-objective calibration of OpenMalaria, using two prior distributions, namely a Gaussian Process (GP) emulator and a *superlearning* algorithm in form of a GP stacked generalization (GPSG) emulator [13].

OpenMalaria captures the events and processes following infection of a human host, simulating malaria infection from mosquitos to individuals and modeling infection characteristics within humans, including parasite densities over time, duration of infection, infectivity to mosquitoes, and importantly the health outcomes due to infections such as anemia, uncomplicated symptomatic malaria, complicated severe malaria with and without co-morbidities, and direct or indirect malaria mortality. The model approximates the level of malaria transmission and epidemiology in a relatively homogeneous setting. OpenMalara simulations are driven by a yearly pattern of force of infection in the absence of interventions. This force of infection is determined by an input entomological inoculation rate (EIR). In the base model used in our analysis, once infected, each simulated infected human host has an associated parasite density time course depending on innate and acquired immunity and duration of infection (modeled individually this allows us to capture disease outcome effects such as immunity acquisition, infectiousness to mosquitoes, morbidity, or mortality). OpenMalaria specifically incorporates between and within host heterogeneity in host exposure, susceptibility, and immune response. The immune dynamics consider the effects of several factors such as acquired and innate immunity, including against severe disease. The model includes a human demography structure and captures seasonality in infection from mosquitoes [14–16]. OpenMalaria also includes a detailed representation of the health system [17] while tracking multiple health outcomes over time (Fig. 1 and Table S1). Monitored health outcomes from the model over time can include *Plasmodium falciparum* age specific prevalence of infections (*Pf*PR), uncomplicated clinical disease

rates, severe disease rates in and out of hospital, and direct or indirect malaria mortality [6–8].

The model allows user specification of detection thresholds for both estimating population-level *Pf*PR and for detection of clinical cases. Diagnostic threshold, sensitivity and specificity can be supplied. In our simulations we assumed a diagnostic threshold of 60 asexual parasites per microliter, with 98% sensitivity and specificity. Small deviations from these assumptions (i.e., parasite threshold between 20 and 150 parasites per microliter and sensitivity/specificity of 85% to 99%) minimally changed the model outputs (data not shown). Also relevant to this work, hospitalization episodes are first defined as those events that would have led to an admission diagnosis of severe malaria, had the patient presented to a health facility. From those episodes, a predefined rate determines the hospitalizations from all expected severe cases. The probability that a clinical malaria episode occurs depends on both the simulated parasite density and a modeled pyrogenic threshold. These episodes include a subset that are severe. These severe episodes can occur as a result of one or other of two distinct processes 1) one subset of the severe malaria episodes comprises those that occur when the host experiences parasitemia over a parasite density threshold. This is modeled as severe episodes arising when a single host- and exposure-independent critical parasite density is exceeded, which is constant over all individuals and time points; 2) the second subset of severe malaria episodes occurs when an otherwise uncomplicated malaria episode happens to coincide with some other insult (e.g., a bacterial infection, malnutrition, or anemia, which occurs with risk conditional *to* the individual age over time. The age profile of the non-malaria insults is modeled as a two-parameter hyperbolic curve. The three parameters of the submodel, the critical parasite density and those defining the hyperbolic function have been previously fitted as described above [15]. The age profile of the non-malaria insults is used as the background comorbidity occurrence for both datasets, parameterized in the contemporary simulations as a proportion of those in the historical simulations. Table S1 summarizes the key model components, processes and assumptions, and references the previous studies where these assumptions have been quantified, fitted, and validated using field epidemiological data.

Supplementary Note 3. *Details on regression models, model selection criteria, computation of prediction intervals and evaluation of predictions*

A regression model framework was implemented to describe the statistical relationship between the scenario modeling outputs, the *Pf*PR$_{2\text{-}10}$ and the malaria hospitalization rates. The framework was based on the previous analyses performed by Paton et al (2022) [3] for a composite sub-set of malaria hospitalizations of severe malaria anemia, cerebral malaria and respiratory distress episodes, and aimed to test whether simulations through OpenMalaria were able to recover the functional relationships obtained from the empirical estimates. The rate of hospital admission denoted for a given simulated time-site i as R_i, was modeled as a negative binomial distribution with probability p and size s parameters $R_i \sim NB\,(p(\lambda_i, r), r)$. We aimed to compare three regression models in predicting the rate of malaria admissions obtained via scenario analyses, as $log(\lambda) \sim f(PfPR_{2-10})$, namely the functional forms of 1) the 3-parameter log-logistic $log(\lambda) = \alpha + \beta \text{ x } PfPR_{2-10}$ and 2) the log-linear $log(\lambda) = \alpha + \beta/(1 + e^{-\gamma\, x\, PfPR_{2-10}})$. Fitting procedure was implemented in R (version 4.0.2). Uncertainty estimates were obtained using a bootstrap procedure described elsewhere [18,19]. For comparison of the models, we used the difference of Akaike Information Criterion (ΔAIC) and Bayesian Information Criterion (ΔBIC) [20], with reduction greater than 10 in favor of the more complex model [3] . Table S2 depicts the obtained parameters and selection criterion values for the key scenarios used in the main text (*historical*, steady-state transmission; *contemporary,* steady-state transmission; and *contemporary*, unsteady transmission) along with the 2 specific functional forms of the regression models. Parametric fits using the log-logistic function showed better performance with simulations under steady-state transmission, both for the historical and contemporary scenarios, while the log-linear function fit better to the simulations based on unsteady transmission.

We further computed the 95% and 50% prediction interval (PI) bounds under the best fit regression models using a bootstrapping procedure [19] for each of the key scenarios. First, we generated a bootstrapped dataset by sampling *n* time-sites randomly with replacement among simulated time-sites within the *Pf*PR$_{2\text{-}10}$ range. Then, we estimated the regression parameters by using the bootstrapped dataset. Finally, we simulated hospitalization rates for all time-sites under our model by using the parameters estimates from the bootstrapped dataset. We repeated the 3 steps 10,000 times to generate 10,000 simulated time-sites computed to the lower and upper PI bounds (PI 2.5%–97.5% and 25%-75%). We smoothed the 95% and 50% PI bounds by using ggplot2 in R. Regression models, evaluation and bootstrapping procedures were

implemented in R (version 4.0.2) and are available at github.com/PDeSalazarSwissTPH/SevereMalaria .

Last, in order to formally test the scenario modeling outputs in recovering the empirical relationship, we contrasted the simulation-based regression models against the empirical estimates. Specifically, we used the log-logistic and log-linear regression models to compute predictions of the hospitalization rates for each of the contemporary time-sites based on the *ePf*$PR_{2\text{-}10}$ for each time-site. We compared predicted vs observed time-site hospitalization rates residuals using evaluation metrics including the root-mean square error (RMSE), root-mean square log error (RMSLE), root square error (RSE) the mean square error (MSE), the mean absolute error (MAE). Fig S9 depicts observed versus predicted contemporary hospitalization rates for each time-site using (a) the log-logistic model, and b) the log-linear model. As seen in Table S3, all performance metrics improved using the log-linear regression model. Model performance evaluation was implemented in R (version 4.0.2) and is available at github.com/PDeSalazarSwissTPH/SevereMalaria.

Supplementary Note 4. *Evaluation of the major epidemiological determinants in the scenario modeling framework*

The model structure of OpenMalaria is based on assumptions given prior scientific evidence and/or empirical observations on malaria transmission dynamics and pathogenesis. Before evaluating the contemporary and the historical empirical datasets, we used a conceptual framework based on causal inference[21] to systematically evaluate key assumptions we needed for designing the simulation scenarios, and to ensure consistency with the major changes that have occurred between the 90s (when the first dataset was collected) and a contemporary time in malaria clinical and epidemiological interventions . First, we defined the core causal pathways of the variables that defined the malaria outcomes, namely exposure, infection (for prevalence) and severe outcomes (for hospitalization incidence). We then defined and included the epidemiological factors -as exposures- that influence the process leading from exposure to infective mosquito bites towards infection and severe disease. We focus on factors that, if present, drive differences at the population level (such as exposure to a certain entomological inoculation rate) in opposition to those that lead to individual variability of severe disease risk (such as individual variability in becoming infected after a mosquito bite). We then formulated a simple regression model for the occurrence of severe disease at the population level as a function of malaria prevalence and included as predictors: 1) the overall immunity of the observed population of children, 2) the case management, which includes effective diagnostic and effective treatment, and 3) the occurrence of comorbidities influencing malaria severe risk. Thus, we assumed that the hospitalization risk among children is a function of the ongoing exposure, the life-long immunity elicited by the previous years exposure, the joint probability of receiving diagnostic and treatment and the efficacy of these diagnostic and treatment and the probability of co-occurring comorbidities. The conceptual framework allowed us to systematically address all the OpenMalaria parameters that relate to these statistical predictors.

Further, we focused on defining scenarios (i.e., simulations over the targeted $Pf\mathrm{PR}_{2\text{-}10}$ range with a specific set of parameters values based on assumptions on the described determinants) that could test the impact of the assumptions in accurately recovering the contemporary relationship between community prevalence and hospitalization rates. We addressed A) the rate of health-care access of uncomplicated and severe malaria (i.e. rate of accurate diagnostic of true occurrence and subsequent timely treatment) B) the efficacy of the available malaria drugs at each period (e.g., the efficacy of artemisinin derivatives combination therapies in clearing malaria), and C) the co-occurrence of other diseases with influence on malaria severe progression over age-groups. For each identified determinant, we implemented a framework which included 3 different scenarios including 1) a *main scenario* parameterized based on data from the literature

2) two more extreme *secondary scenarios* (i.e., stronger assumption) parameterized using values above and below the range of those included from the literature. Specifically, we evaluated the following: A) main scenario: access rates of malaria requiring hospitalization have ranged from 60%-90% while the access rates of uncomplicated malaria treatment ranged 20%-40% based on the higher estimates from the Demographic and Health Surveys program [22]; secondary scenario 1: health care access rates have been substantially lower, with hospitalizations being between 40% and 60% and uncomplicated malaria treatment rates of 20%-40%, similar to those estimates in the 1990s; secondary scenario 2: access rates were 90-98%; B) *main scenario*: drug efficacy at the individual level has ranged between 85%-98% given the wide deployment of artemisinin-based combination therapies in the countries [23,24]; and C) *main scenario:* co-occurrence of diseases contributing to malaria hospitalization was substantially reduced by 20%-40% compared to the historical data under the assumption that lower-respiratory tract infections [25] and diarrhea [26], which have reduced substantially their incidence in the targeted countries, are the major contributors; *secondary scenario 1*: drug efficacy has ranged between substantially lower values, with expectation of 50%; *secondary scenario 2*: drug efficacy is 98-100%; *secondary scenario 1:* comorbidities occurrence has been similar to those estimated during the 90s; *secondary scenario 2:* comorbidities contributing to hospitalization rates were reduced by 75%-90%.

Overall, the different set of parameterizations aimed to be consistent with the following characteristics of the contemporary empirical dataset: a) malaria population-based hospitalization estimates were aligned with DHS estimates [22], and b) individuals with primarily reported comorbidities that were not malaria had been excluded in their majority from the analysis dataset.

We also performed a sensitivity evaluation of the empirical prevalence estimates by time-site by comparing the estimates to those obtained using a geospatial model. Modeled $mPfPR_{2\text{-}10}$ estimates were explicitly obtained for each time-site catchment population using a geospatial model detailed elsewhere [27,28], a Bayesian hierarchical geostatistical framework based on more than 180000 geo-coded empirical prevalence survey data points from East Africa, interpolated in time to 1 x 1 km resolutions using climatic and ecological covariates. As seen in fig S10, the modeled estimates, computed for the time period when the hospitalization estimates were obtained, reproduce the overall “exponential” relationship with only one time-site showing a significant deviation (fig S10 A). Consistently the correlation between the mPfPR and the ePfPR shows a linear relationship (fig S10 B).

Supplementary Note 5. *Recovery of hospitalization risk by age group*

We further explored the implications of time-varying transmission on the age-structure of the hospitalization rates. To assess the performance of the modeling framework in recovering the disease risk over age groups, we compared both empirical and simulation-based estimates disaggregated by one year age-groups, except for those between 3 months and 2 years which were all aggregated in a single group for simplicity as empirical estimates for children under 3 months were not available. We then performed the 4-steps iterative analysis for the present-day dataset under the steady and time-varying transmission assumptions as previously described and evaluated the $Pf\mathrm{PR}_{2\text{-}10}$ -severe disease incidence relationship by age-group. As a case study, we computed the resulting hospitalization incidence over age-groups at values of $Pf\mathrm{PR}_{2\text{-}10}$ equal to those estimated in the four representative time-sites Apac A, Busia, Mubende B and Jinja B.

Age-structure of malaria severe disease under steady transmission is known to show characteristic dynamics, with low prevalence settings shifting the severe cases towards older children relative to higher transmission settings [29]. As a result, severe malaria incidence among young and very young children in high transmission settings are typically higher than among same age-groups in low transmission settings whereas the opposite occurs among older children. When we examine the empirical patterns disaggregated by age-group (fig S2), we observed that the exponential relationship present in the younger ages is maintained over the older groups, although with less steepness. This is in opposition to what would be observed in clinical series with an asymptotic pattern (i.e., with steady transmission), as the relationship among older children would transition towards an asymptotic pattern (or even a convex one).

The simulations under the time-varying transmission assumption reasonably recover the trends across age-groups. Consistent with the main results here, we find that, for any age group, severe incidence increases towards higher $Pf\mathrm{PR}_{2\text{-}10}$. Further, the empirical data shows that time sites with low $Pf\mathrm{PR}_{2\text{-}10}$ present a lower-than-expected severe malaria risk for older ages. When analyzing these trends by looking at the incidence over age-groups disaggregated by time-site (fig. S3, plotting 4 representative time-sites) the empirical estimates of severe malaria occurrence over age-groups (left column) are better recovered under the time-varying assumption (center column) than under the steady- state transmission assumption. The overall analysis is consistent with an excess (e.g., fig. S3, Apac A) or reduction (e.g. fig. S3, Jinja B) of severe disease risk relative to the steady-state assumption, particularly towards older ages, as expected with time-varying exposure and subsequent gap between age-dependent developed immunity and transmission at assessment.

Fig. S1.

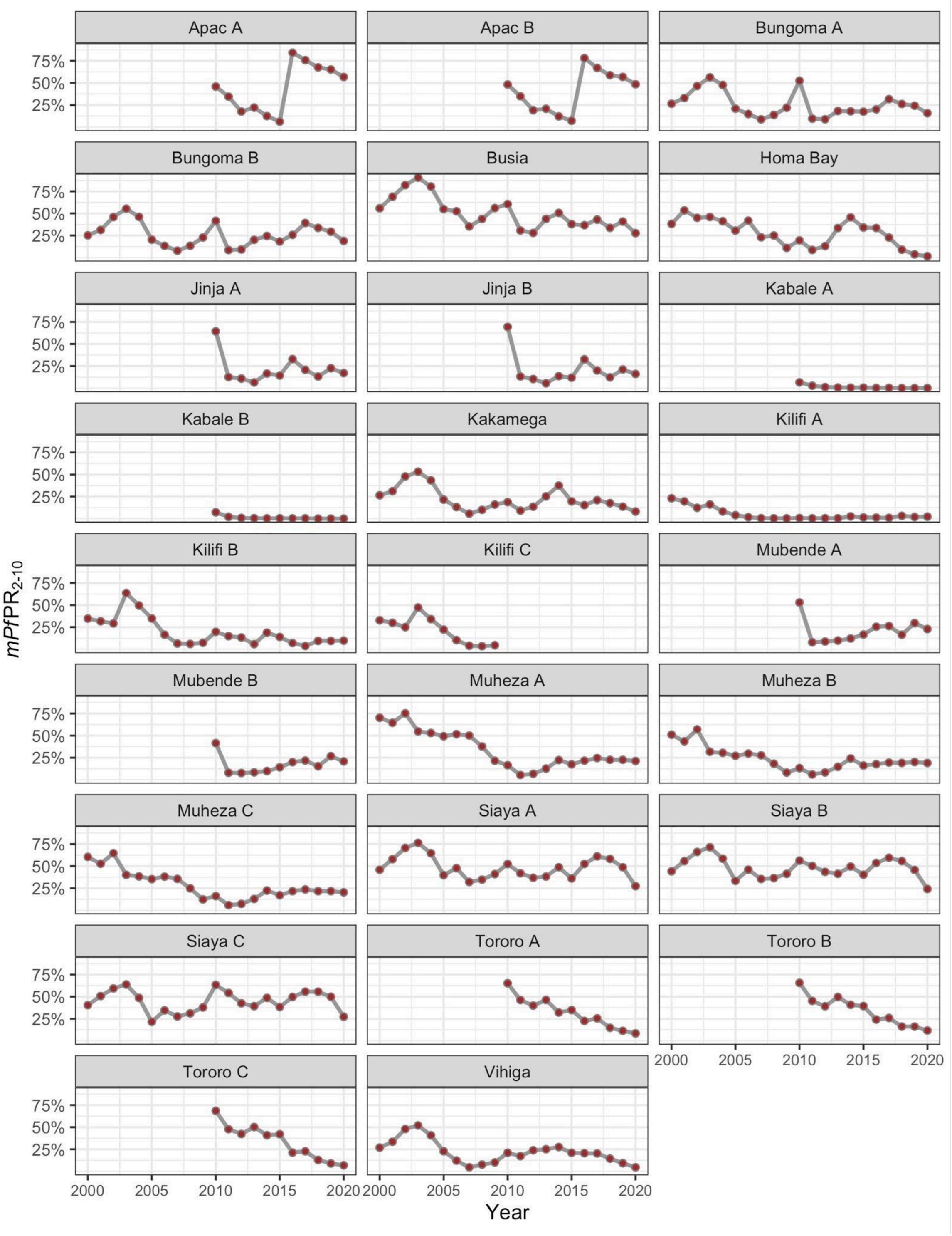

Fig. S1. Available modeled *Pf*PR$_{2\text{-}10}$ time series estimates between 2000-2020 for all sites included in the East-African contemporary dataset obtained from Bayesian hierarchical geospatial models [27,28] (site indicated in plot title).

Fig. S2.

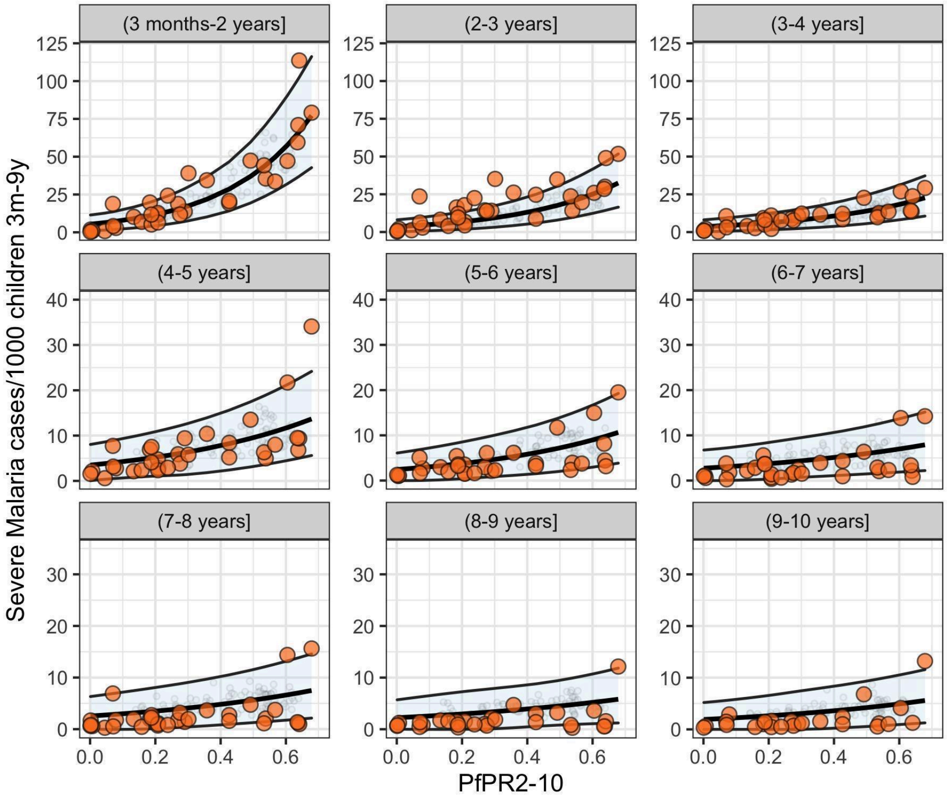

Fig. S2. ***Recovery of the empirical Pf*****PR**$_{2\text{-}10}$***-hospitalization incidence relationship over age groups and under unsteady transmission.*** Plots show the empirical *Pf*PR$_{2\text{-}10}$-hospitalization incidence estimates by age group obtained from the contemporary dataset (orange dots) overlapping the modeled *Pf*PR$_{2\text{-}10}$-severity estimates obtained through simulations under the time-varying transmission assumption (gray dots) and the subsequent log-linear regression model (median and 95% prediction intervals, black lines).

Fig. S3.

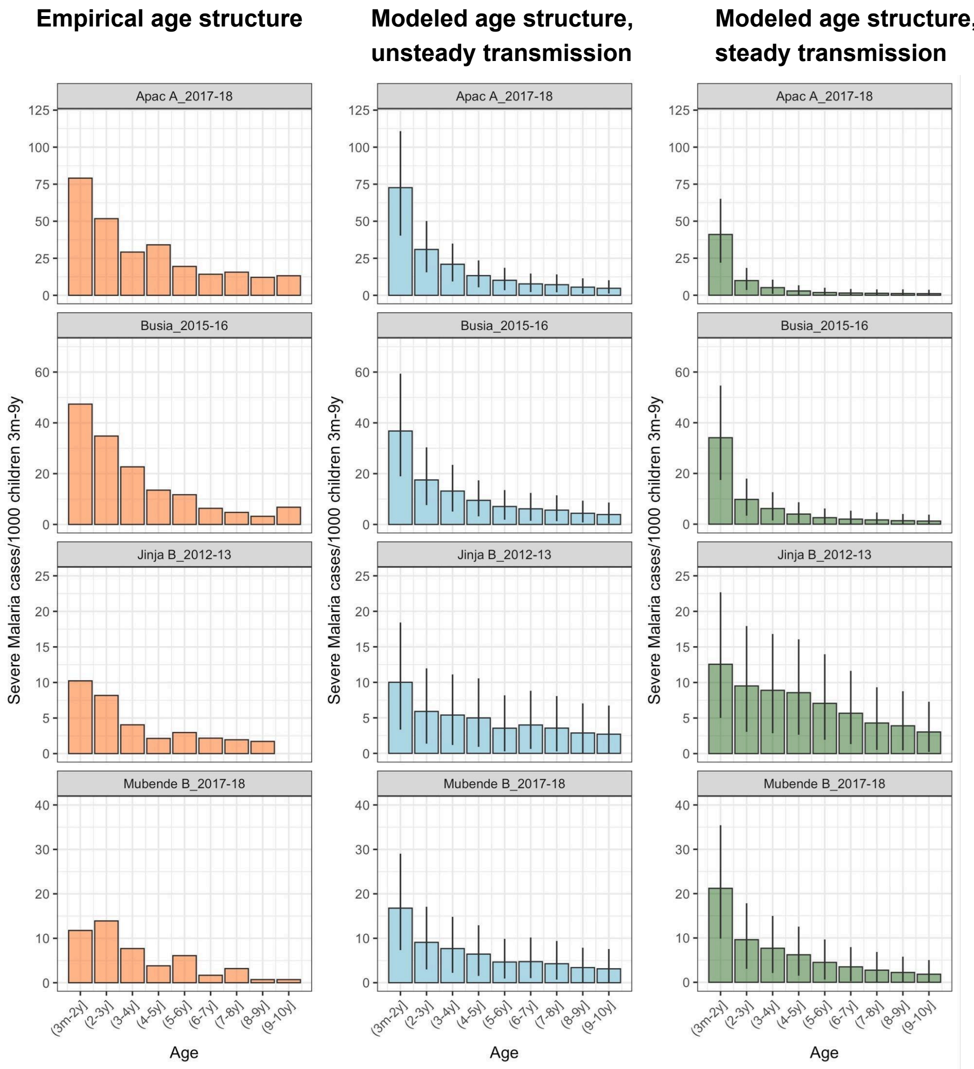

Fig. S3. ***Comparison of the empirical age structure of malaria hospitalization rates with unsteady and steady transmission modeled estimates.*** Depiction of the empirical age structure of malaria hospitalizations for children 3 months to 9 years old (left column, orange) and modeled age structure computed under the steady exposure assumption (middle column, blue) and unsteady exposure (right column, green) age structure in four representative time sites (indicated in plot titles).

Fig. S4.

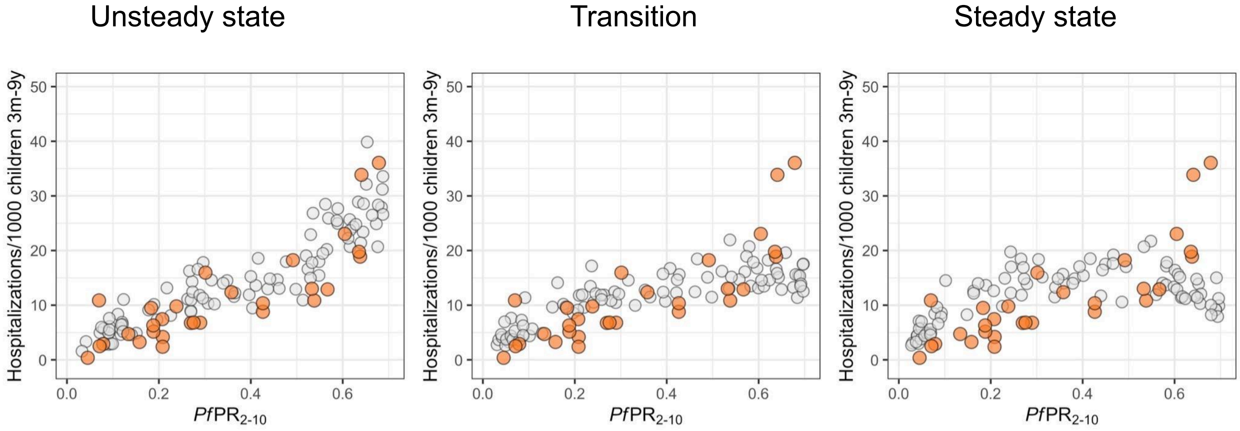

Fig. S4. Depicting the prevalence-hospitalization relationship obtained through simulations (gray points) under unsteady exposure, a transition stage towards equilibrium, and under steady exposure. Orange points showing the empirical relationship for comparison at each stage.

Fig. S5.

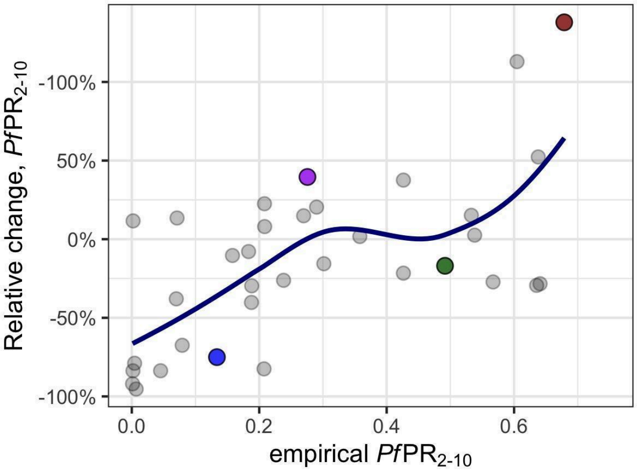

Fig. S5. Scatterplot depicting the relationship between empirical community prevalence as empirical *Pf*PR$_{2\text{-}10}$, and estimated relative change at survey (as %) computed from the median value of the modeled *Pf*PR$_{2\text{-}10}$ of the past 7-9 years before empirical survey for each time-site. Four representative time-sites are highlighted, Apac A (red), Busia (green), Jinja B (purple) and Mubende B (blue) colored dots). Blue line shows the best fit using a nonparametric local polynomial regression suggesting a quasilienar trend in the relationship.

Fig. S6.

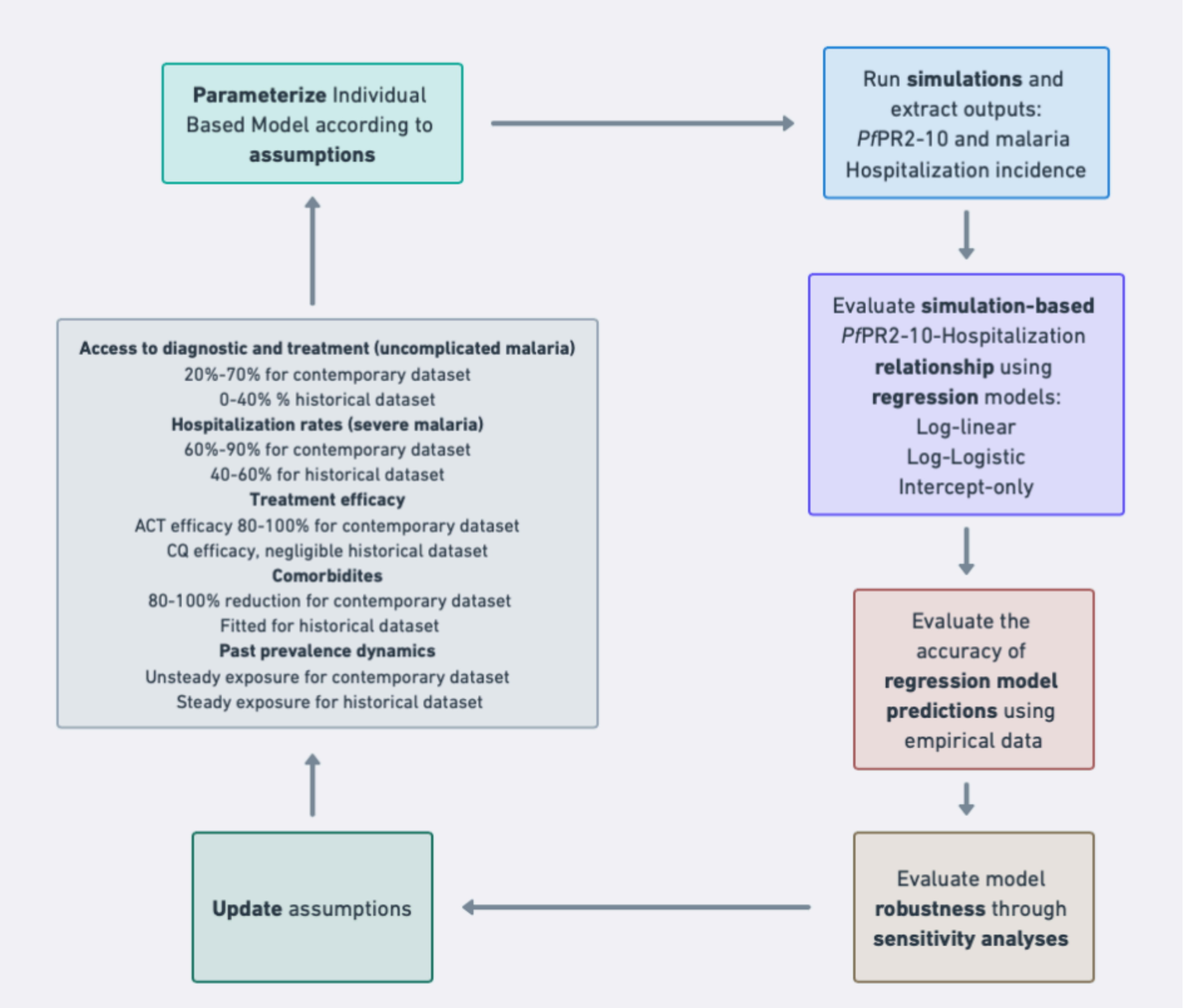

Fig. S6. Schematic illustration of the iterative analysis approach to interrogating empirical data with mechanistic models

Fig. S7.

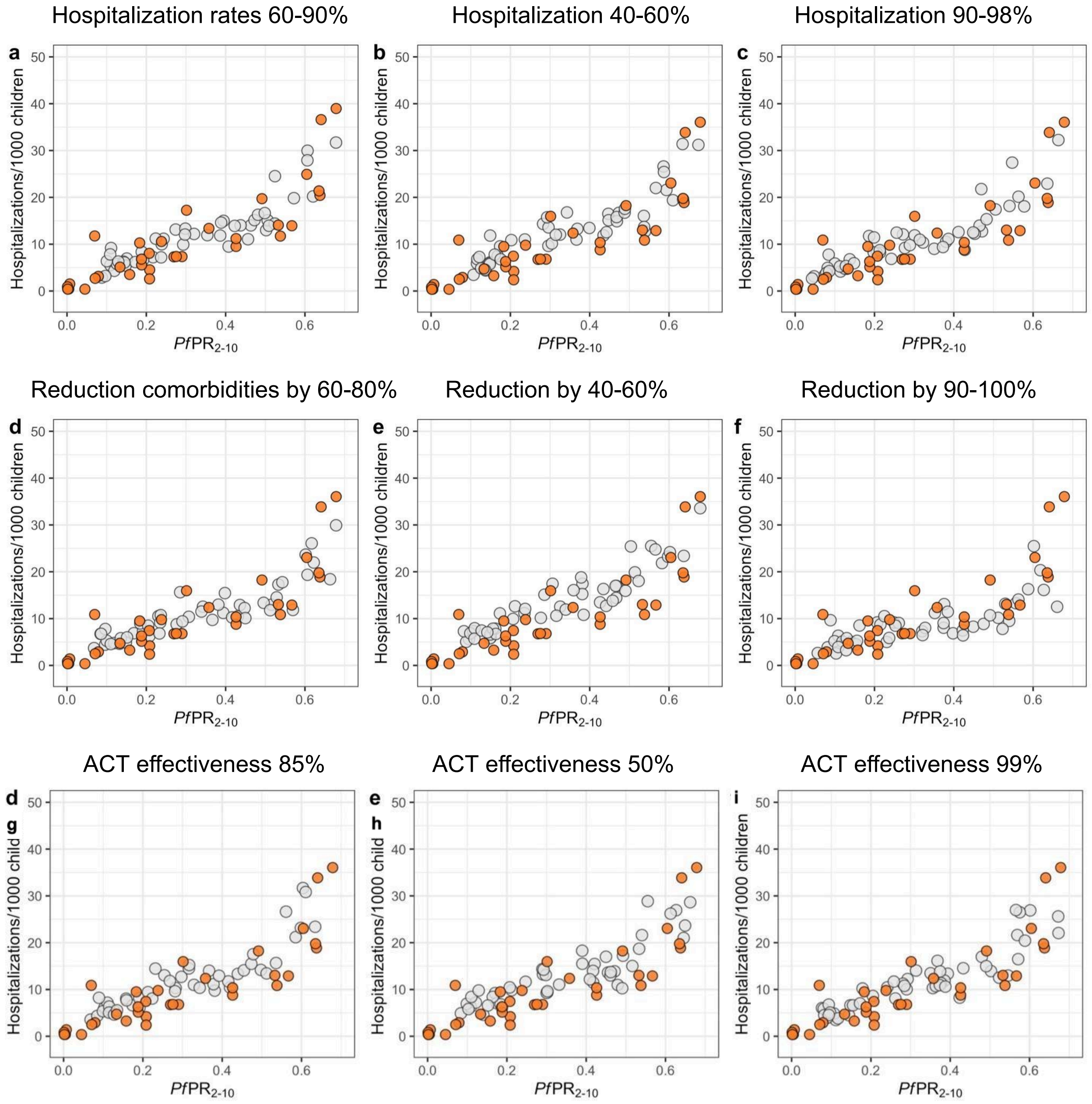

Fig. S7 Results of the sensitivity analysis of our main model parameterization assumptions under unsteady transmission. a-c) Sensitivity to access to hospital care for severe disease, **d-f)** Sensitivity to levels of comorbidities, **g-i)** Sensitivity to access and effectiveness of first-line AC treatment of uncomplicated malaria.

Fig S8.

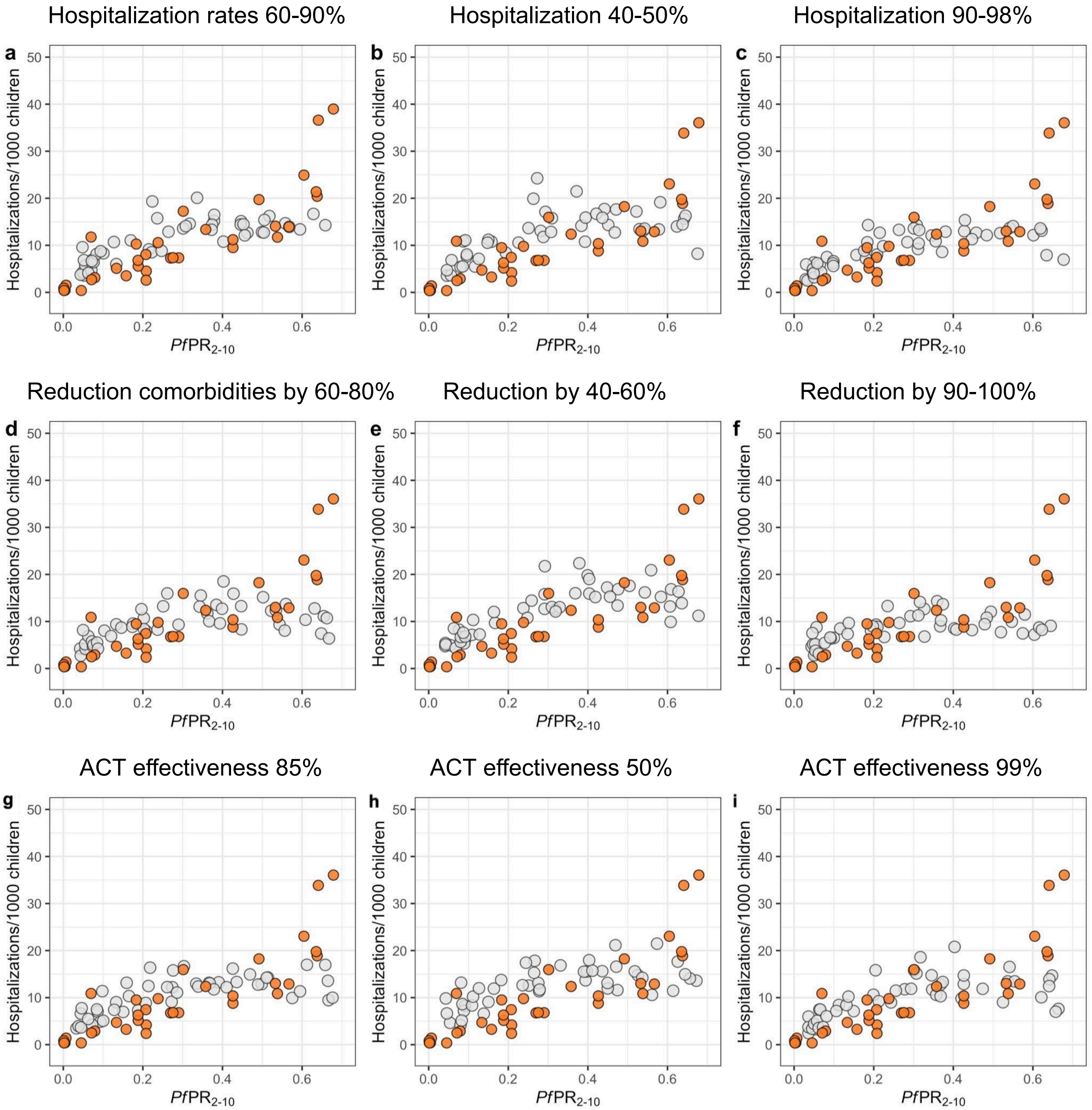

Fig. S8 Results of the sensitivity analysis of our main model parameterization assumptions under steady transmission. a-c) Sensitivity to access to hospital care for severe disease, **d-f)** Sensitivity to levels of comorbidities, **g-i)** Sensitivity to access and effectiveness of first-line AC treatment of uncomplicated malaria.

Fig S9.

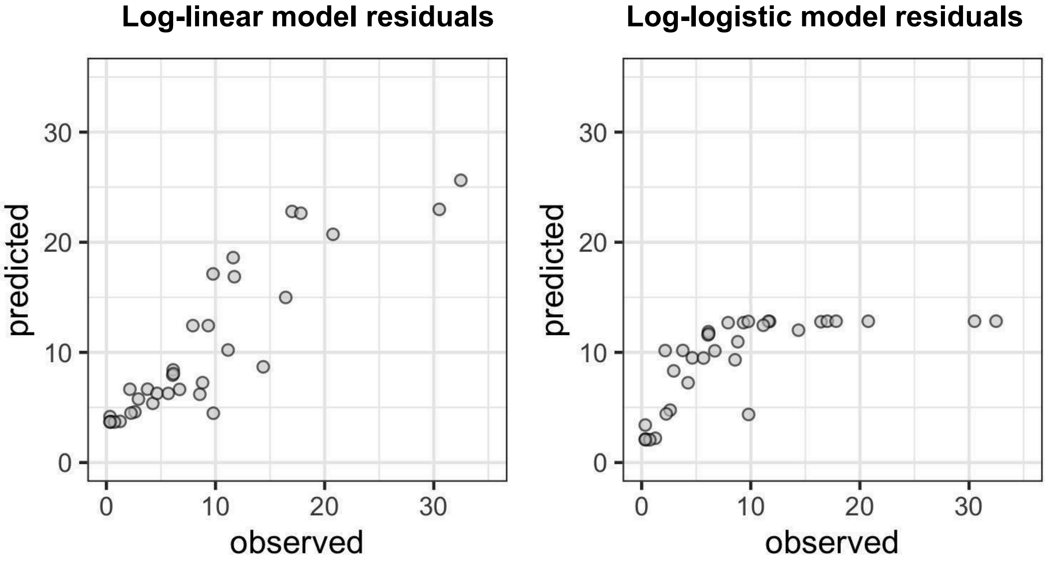

Fig. S9. Comparison of residuals from the regression-based predicted *Pf*PR$_{2\text{-}10}$ versus observed empirical *Pf*PR$_{2\text{-}10}$.

Fig S10

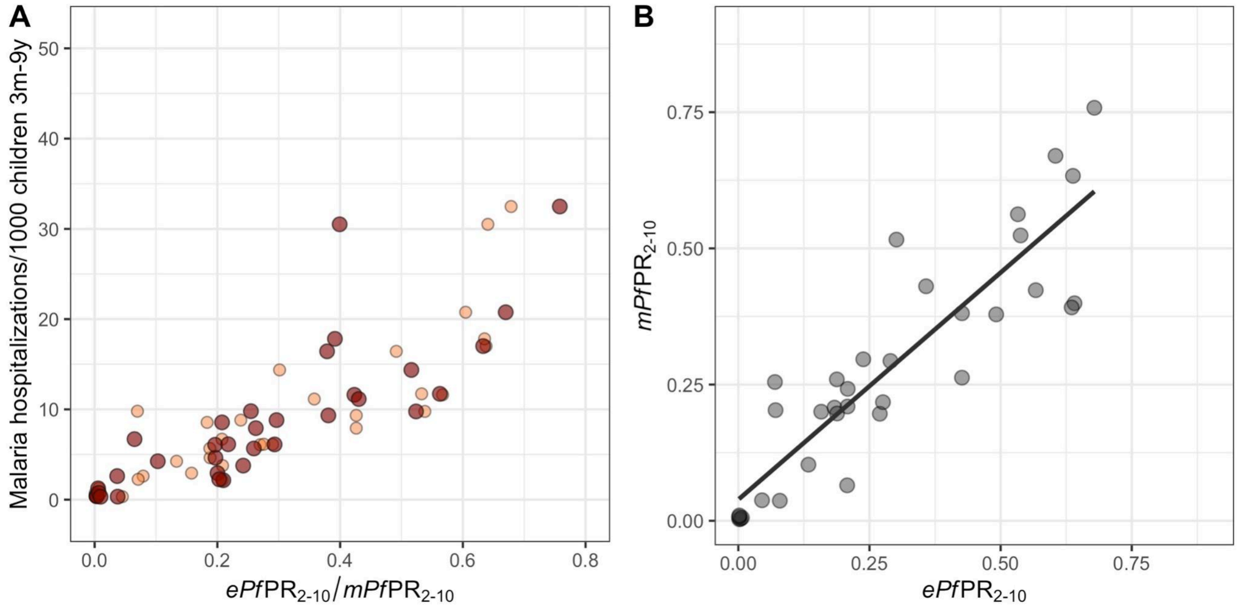

Fig. S10. (A) Comparison of the prevalence-hospitalization relationship obtained using the empirical *Pf*PR$_{2\text{-}10}$(orange) and the modeled *Pf*PR$_{2\text{-}10}$ (red) computed for the specific catchment populations and time-periods of the hospitalization data. (B) Correlation between the empirical and the modeled *Pf*PR$_{2\text{-}10}$ for the same time-sites (gray) and linear relationship (black line).

Table S1 Summary of the OpenMalaria model components (adapted from [30])

Name and reference/s	Description of core assumptions
Key modeled epidemiological processes (base model)	
Malaria infection of humans [12] also detailed in eq. 1-4 of Additional file 1 in [8]	● Determined by EIR which is a model input and affects the force of infection in the simulated setting.<br>● The model includes functions that allow age-dependent exposure of human hosts to mosquitoes (correlating with body-surface area).<br>● The relationship between infection rates and EIR was determined by fitting to data from The Gambia, Nigeria and Kenya in [12].
Infection progression in humans: asexual parasite densities and immunity [16,31,32] and eq. 5-15 of Additional file 1 in [8]	● In the base model. blood-stage parasite density depends on the time since infection and is affected by naturally acquired immunity. Acquired immunity reduces parasite density of subsequent infections.<br>● The duration of infection follows a log-normal distribution and was estimated from a malaria therapy dataset ([16,31] and eq. 1 in [32]<br>● Immunity (both pre-erythrocytic and blood-stage) is host specific and depends on consequent episodes of exposure to infection and total parasitemia seen by an individual in their lifetime.<br>● Super-infection is possible with cumulative parasite densities<br>● The parasite density in a host at a given time is defined and fitted with data from Ghana, Nigeria and Tanzania in [32]
Transmission from infected humans to mosquitoes [16,30,31] and eq. 16-21 of Additional file 1 in [8]	● Infectivity to mosquitoes depends on the density of parasites present in the human (and includes a time-lag for gametocyte development)<br>● The fraction of resulting infected mosquitoes after feeding on a human host follows a binomial distribution.<br>● The relationship between infectivity to mosquitoes and parasite density was informed by data in ([16,31] and with data from malaria therapy collected in Georgia between 1940 and 1963 and available from [31]<br>● The age-specific contribution to overall infectiousness to mosquitoes was also validated against field data collected from Liberia, The Gambia, Tanzania, Kenya, Papua New Guinea and Cameroon.
Disease progression: uncomplicated, severe morbidity, mortality, and anemia.<br><br>[16,33] and eq. 22-32 of Additional file 1 in [8]	● Acute clinical illness depends on the current parasite densities in a human host and their innate pyrogenic threshold. This threshold is dynamic over time and depends on the individual exposure history.<br>● Acute morbidity episodes are either uncomplicated or evolve to severe episodes; A proportion of the severe episodes leads to deaths.<br>● Clinical malaria patterns were fitted with data from Senegal in [16].<br>● The probabilities that a clinical episode will become severe (and the associated risk of mortality for a severe episode) are defined were originally fitted to field data from over 10 African countries in [15]. Some of which are shown in historical data of Fig 1 in the main text, including Bakau, The Gambia (1991-94), Snow et al. (1997) [1]; Kilifi Township, Kenya (1993-96), Robert Snow unpublished data; Foni Kansala, The Gambia (1994-95), Geisler Schnieder and Robert Snow, unpublished data; Sukuta, The Gambia; Kilifi North Kenya (1990-95: Snow et al., (1997) [1]; and Mponda, Malawi (1994-95), Slutsker et al. (1994) [5].
Modeled characteristics of the transmission setting	

Population age structure [15,34]	• Can be supplied by the user. In this study informed by the site in Ifakara, Tanzania, (data available through the INDEPTH network [34].
Transmission seasonality [12,35]	• Force of infection input into the model via EIR, is seasonally forced. The same transmission pattern is reproduced each year in absence of interventions. Users can define any patterns as needed.
Case management [17]	• Treatment seeking and care (including drug efficacy, adherence, etc) of uncomplicated malaria is modeled through a comprehensive decision tree-based model defined and validated in [17]. This model determines the outcomes treatment depending on the occurring clinical events such as fevers and seeking of care<br>• The model includes specification of access to official or non-official care, access to hospital for severe cases, diagnostic tests (use, specificity, sensitivity, and threshold of detection), treatments for first, second line and non-official care, effects of treatment, case fatality rate, case sequelae and cure rates.
Simulation regimes and model variants	
Time steps	- Simulation outputs are tracked every 5 days
Model variants [7]	• Up to 14 model variants are available, including the base model. These variants include distinct assumptions on immunity decay, treatment and heterogeneity of transmission. In this study, we use the base model (parameterization described in Table 2 in [7] under the denomination R0001)
Software availability and documentation	
Source code and wiki page available on GitHub: https://github.com/SwissTPH/openmalaria/	

Table S2.

**Scenario**	**Functional form**	**Selection criterion**
Historical scenario, steady-state transmission	log-logistic	AIC=462; BIC=473
	log-linear	AIC=595; BIC=602
Contemporary scenario, steady-state transmission	log-logistic	AIC=511; BIC=516
	log-linear	AIC=601; BIC=606
Contemporary scenario, unsteady transmission	log-logistic	AIC=509; BIC=514
	log-linear	AIC=483; BIC=488

Table S2. Showing Information Criteria values used for regression model selection. The log-logistic model is favored for simulations under steady-state transmission for both the historical and contemporary scenarios. The log-linear model favored for simulations under unsteady transmission for the contemporary scenario.

Table S3. Performance metrics of the parametric regression models on predicting the empirical relationship

**Metric**	**Log-logistic**	**Log-linear**
RMSE	5.99	3.89
RMSLE	0.598	0.590
RSE	0.58	0.24
MSE	35.9	15.1
MAE	4.4	3.3

Table S4. Data sourced from Paton et al. (2021)[3]

Site, dates	Dates Community (CS) or School Survey (SS)*	Positive/examined [Age range, years]	*Pf*PR2-10 % [95% CI]**	Citation
Kilifi A, Kenya<br>2007-09<br>2010-15<br>2017-19	2007-09 (CS)<br>2010-15 (CS)<br>2017-19 (CS)	10/1645 [0.5-14.9]<br>1/1439 [0.5-14.9]<br>0/355 [0.5-14.9]	0.68 [0.32, 1.12]<br>0.12 [0, 0.34]<br>0.2 [0, 0.87]	36,37
Kilifi B, Kenya<br>2007-09<br>2010-15<br>2018-19	2007-09 (CS)<br>2010-15 (CS)<br>2017-19 (CS)	406/2061 [0.5-14.9]<br>404/2261 [0.5-14.9]<br>62/841 [0.5-14.9]	20.75 [18.96, 22.55]<br>18.82 [17.2, 20.5]<br>7.91 [6.09, 9.84]	36,37
Kilifi C, Kenya<br>2018-19	2018-19 (CS)	267/1336 [0.5-14.9]	**15.78 [13.9, 17.66]**	38
Siaya A, Kenya<br>2010-13	2010-13 (CS)	234/454 [0.1-14.9]	53.81 [49.12, 58.65]	38
Siaya B, Kenya<br>2010-13	2010-13 (CS)	588/1155 [0.1-14.9]	53.29 [50.3, 56.33]	39
Siaya C, Kenya<br>2010-13	2010-13 (CS)	492/808 [0.1-14.9]	63.79 [60.26, 67.2]	39
Busia, Kenya<br>2015-16<br>2017-20	2014 (SS)<br>2019 (SS)	330/596 [4-14.9]<br>285/681 [4-14.9]	**49.16 [45.01, 53.28]**<br>**35.79 [32.22, 39.34]**	3
Kakamega, Kenya<br>2015-16<br>2017-18	2014 (SS)<br>2018-19 (SS)	65/198 [4-14.9]<br>204/789 [4-14.9]	**26.97 [21.25, 33.09]**<br>**20.82 [18.14, 23.59]**	3
Vihiga, Kenya<br>2017-18	2018-19 (SS)	56/596 [4-14.9]	**7.1 [5.3, 9.09]**	3
Bungoma A, Kenya<br>2019-20	2019 (SS)	77/297 [4-14.9]	**20.85 [16.63, 25.36]**	3
Bungoma B, Kenya<br>2019-20	2019 (SS)	137/392 [4-14.9]	**29 [24.69, 33.4]**	3
Homa Bay, Kenya<br>2019-20	2019 (SS)	24/397 [4-14.9]	**4.52 [2.81, 6.44]**	3
Jinja A, Uganda<br>2017-18	2019 (SS)	68/400 [5-16.9]	18.33 [14.46, 22.29]	40

Jinja B, Uganda<br>2012-13<br>2017-18	2012-13 (CS)<br>2019 (SS)	80/637 [0.5-14.9]<br>59/400 [5-16.9]	13.36 [10.8, 16.18]<br>15.8 [12.26, 19.64]	40,41
Tororo A, Uganda<br>2012-13<br>2017-19	2011-13 (CS)<br>2019 (SS)	501/820 [0.1-14.9]<br>27/425 [5-16.9]	64.08 [60.7, 67.63]<br>6.98 [4.64, 9.64]	40,41
Tororo B, Uganda<br>2012-13<br>2017-19	2011-13 (CS)<br>2019 (SS)	303/499 [0.1-14.9]<br>70/399 [5-16.9]	63.54 [59.05, 67.94]<br>18.78 [14.93, 22.86]	40,41
Tororo C, Uganda<br>2012-13	2011-13 (CS)	994/1836 [0.1-14.9]	56.7 [54.29, 59.05]	42
Apac A, Uganda<br>2017-18	2019 (SS)	127/199 [5-16.9]	67.87 [60.75, 74.95]	43
Apac B, Uganda<br>2017-18	2019 (SS)	167/294 [5-16.9]	60.46 [54.34, 66.4]	43
Mubende A, Uganda<br>2017-18	2019 (SS)	77/195 [5-16.9]	42.63 [35.56, 49.9]	43
Mubende B, Uganda<br>2017-18	2019 (SS)	53/208 [5-16.9]	27.57 [21.56, 33.99]	43
Kabale A, Uganda<br>2017-18	2019 (SS)	0/400 [5-16.9]	0.18 [0, 0.79]	43
Kabale B, Uganda<br>2017-18	2019 (SS)	1/400 [5-16.9]	0.44 [0.01, 1.25]	43
Muheza A, Tanzania<br>2006-07	2008 (CS)	13/39 [0.1-4.9]	**30.14 [18.08, 42.53]**	44
Muheza B, Tanzania<br>2006-07	2008 (CS)	164/671 [0.1-99.0]	**23.82 [20.41, 27.37]**	45
Muheza C, Tanzania<br>2006-07	2008 (CS)	417/1050 [0.4-19.9]	42.63 [39.44, 45.79]	45

*At each site, diverse sampling strategies were implemented. In Siaya A-C and Kilifi A-B, households underwent annual sampling as part of long-term surveillance. Kilifi C saw four rounds of household sampling, aligning with the hospital surveillance period. School surveys were conducted in various regions of Kenya (Busia, Kakamega, Vihiga, Bungoma, Homa Bay) and Uganda (Jinja A, Jinja B 2017-2018, Tororo A 2017-2019, Tororo B 2017-2019, Apac A & B, Mubende A & B, Kabale A & B). The survey included all public, primary schools within hospital catchment areas, with annual community-based household surveys also taking place. Community surveys in Jinja B and Tororo A and C (2012-2013) were part of broader household sample surveys across respective districts, with data limited to catchment parishes. Published findings in Muheza, Tanzania, provided village-level data reflecting time-site transmission estimates. Due to the varied sampling methods, the incorporation of sampling weights in

PfPR$_{2\text{-}10}$ estimation was precluded. However, at all sites (excluding Muheza), samples covered entire community or school catchment areas within selected hospital catchment areas.

**Parasite prevalence, adjusted for the 2-10 age range as detailed in methods is presented in bold. Estimates conducted by RDT were corrected to microscopy values using a regression framework outlined in Mappin et al. (2015)[46].

References:

1. Snow, R. W. *et al.* Relation between severe malaria morbidity in children and level of Plasmodium falciparum transmission in Africa. *The Lancet* vol. 349 1650–1654 Preprint at https://doi.org/10.1016/s0140-6736(97)02038-2 (1997).
2. Marsh, K. & Snow, R. W. Malaria transmission and morbidity. *Parassitologia* **41**, (1999).
3. Paton, R. S. *et al.* Malaria infection and severe disease risks in Africa. *Science* **373**, (2021).
4. Smith, D. L., Guerra, C. A., Snow, R. W. & Hay, S. I. Standardizing estimates of the Plasmodium falciparum parasite rate. *Malar. J.* **6**, 1–10 (2007).
5. Slutsker, L., Taylor, T. E., Wirima, J. J. & Steketee, R. W. In-hospital morbidity and mortality due to malaria-associated severe anaemia in two areas of Malawi with different patterns of malaria infection. *Trans. R. Soc. Trop. Med. Hyg.* **88**, (1994).
6. Smith, T. *et al.* Towards a comprehensive simulation model of malaria epidemiology and control. *Parasitology* **135**, 1507–1516 (2008).
7. Smith, T. *et al.* Ensemble modeling of the likely public health impact of a pre-erythrocytic malaria vaccine. *PLoS Med.* **9**, e1001157 (2012).
8. Smith, T. *et al.* Mathematical modeling of the impact of malaria vaccines on the clinical epidemiology and natural history of Plasmodium falciparum malaria: Overview. *Am. J. Trop. Med. Hyg.* **75**, 1–10 (2006).
9. Penny, M. A. *et al.* Distribution of malaria exposure in endemic countries in Africa considering country levels of effective treatment. *Malar. J.* **14**, 384 (2015).
10. Penny, M. A. *et al.* Public health impact and cost-effectiveness of the RTS,S/AS01 malaria vaccine: a systematic comparison of predictions from four mathematical models. *Lancet*

387, 367–375 (2016).

11. Smith, N. R. *et al.* Agent-based models of malaria transmission: a systematic review. *Malar. J.* **17**, 299 (2018).
12. Smith, T. *et al.* Relationship between the entomologic inoculation rate and the force of infection for Plasmodium falciparum malaria. *Am. J. Trop. Med. Hyg.* **75**, (2006).
13. Reiker, T. *et al.* Emulator-based Bayesian optimization for efficient multi-objective calibration of an individual-based model of malaria. *Nat. Commun.* **12**, 7212 (2021).
14. Ross, A., Killeen, G. & Smith, T. Relationships between host infectivity to mosquitoes and asexual parasite density in Plasmodium falciparum. *Am. J. Trop. Med. Hyg.* **75**, 32–37 (2006).
15. Ross, A., Maire, N., Molineaux, L. & Smith, T. An epidemiologic model of severe morbidity and mortality caused by Plasmodium falciparum. *Am. J. Trop. Med. Hyg.* **75**, 63–73 (2006).
16. Smith, T. *et al.* An epidemiologic model of the incidence of acute illness in Plasmodium falciparum malaria. *Am. J. Trop. Med. Hyg.* **75**, 56–62 (2006).
17. Tediosi, F. *et al.* An approach to model the costs and effects of case management of Plasmodium falciparum malaria in sub-saharan Africa. *Am. J. Trop. Med. Hyg.* **75**, 90–103 (2006).
18. De Salazar, P. M., Cox, H., Imhoff, H., Alexandre, J. S. F. & Buckee, C. O. The association between gold mining and malaria in Guyana: a statistical inference and time-series analysis. *Lancet Planet Health* **5**, e731–e738 (2021).
19. De Salazar, P. M., Niehus, R., Taylor, A., Buckee, C. O. & Lipsitch, M. Identifying Locations with Possible Undetected Imported Severe Acute Respiratory Syndrome Coronavirus 2 Cases by Using Importation Predictions. *Emerg. Infect. Dis.* **26**, 1465 (2020).
20. Dziak, J. J., Coffman, D. L., Lanza, S. T., Li, R. & Jermiin, L. S. Sensitivity and specificity of information criteria. *Brief. Bioinform.* **21**, (2020).
21. Hernan, M. A. & Robins, J. M. *Causal Inference*. (CRC Press, 2019).

22. The DHS Program. https://dhsprogram.com/methodology/survey-types/mis.cfm.

23. World Health Organization. *Guidelines for the Treatment of Malaria*. (World Health Organization, 2010).

24. World Health Organization. *Guidelines for the Treatment of Malaria. Third Edition*. (World Health Organization, 2015).

25. Reiner, R. C. *et al.* Identifying residual hotspots and mapping lower respiratory infection morbidity and mortality in African children from 2000 to 2017. *Nat Microbiol* **4**, 2310–2318 (2019).

26. Reiner, R. C., Jr *et al.* Variation in Childhood Diarrheal Morbidity and Mortality in Africa, 2000-2015. *N. Engl. J. Med.* **379**, 1128–1138 (2018).

27. Kamau, A. *et al.* Malaria hospitalisation in East Africa: age, phenotype and transmission intensity. *BMC Med.* **20**, 28 (2022).

28. Alegana, V. A. *et al.* Plasmodium falciparum parasite prevalence in East Africa: Updating data for malaria stratification. *PLOS Global Public Health* vol. 1 e0000014 Preprint at https://doi.org/10.1371/journal.pgph.0000014 (2021).

29. Carneiro, I. *et al.* Age-patterns of malaria vary with severity, transmission intensity and seasonality in sub-Saharan Africa: a systematic review and pooled analysis. *PLoS One* **5**, e8988 (2010).

30. Golumbeanu, M. *et al.* Leveraging mathematical models of disease dynamics and machine learning to improve development of novel malaria interventions. *Infect Dis Poverty* **11**, 61 (2022).

31. Collins, W. E. & Jeffery, G. M. A retrospective examination of the patterns of recrudescence in patients infected with Plasmodium falciparum. *Am. J. Trop. Med. Hyg.* **61**, 44–48 (1999).

32. Maire, N. *et al.* A model for natural immunity to asexual blood stages of Plasmodium falciparum malaria in endemic areas. *Am. J. Trop. Med. Hyg.* **75**, (2006).

33. Ross, A. & Smith, T. The effect of malaria transmission intensity on neonatal mortality in

endemic areas. *Am. J. Trop. Med. Hyg.* **75**, (2006).

34. Ekström, A. M. *et al.* INDEPTH Network: contributing to the data revolution. *The lancet. Diabetes & endocrinology* **4**, (2016).

35. Stuckey, E. M., Smith, T. & Chitnis, N. Seasonally dependent relationships between indicators of malaria transmission and disease provided by mathematical model simulations. *PLoS Comput. Biol.* **10**, e1003812 (2014).

36. Muthui, M. K. *et al.* Gametocyte carriage in an era of changing malaria epidemiology: A 19-year analysis of a malaria longitudinal cohort. *Wellcome Open Research* **4**, (2019).

37. Wamae, K. *et al.* Transmission and Age Impact the Risk of Developing Febrile Malaria in Children with Asymptomatic Plasmodium falciparum Parasitemia. *J. Infect. Dis.* **219**, 936 (2019).

38. Kamau, A. *et al.* Malaria infection, disease and mortality among children and adults on the coast of Kenya. *Malar. J.* **19**, 1–12 (2020).

39. Were, V. *et al.* Trends in malaria prevalence and health related socioeconomic inequality in rural western Kenya: results from repeated household malaria cross-sectional surveys from 2006 to 2013. *BMJ Open* **9**, (2019).

40. Irimu, G. *et al.* Approaching quality improvement at scale: a learning health system approach in Kenya. *Arch. Dis. Child.* **103**, (2018).

41. Kamya, M. R. *et al.* Malaria transmission, infection, and disease at three sites with varied transmission intensity in Uganda: implications for malaria control. *Am. J. Trop. Med. Hyg.* **92**, (2015).

42. Staedke, S. G. *et al.* The PRIME trial protocol: evaluating the impact of an intervention implemented in public health centres on management of malaria and health outcomes of children using a cluster-randomised design in Tororo, Uganda. *Implement. Sci.* **8**, 1–13 (2013).

43. Mpimbaza, A. *et al.* The age-specific incidence of hospitalized paediatric malaria in

Uganda. *BMC Infect. Dis.* **20**, (2020).

44. Ishengoma, D. S. *et al.* Trends of Plasmodium falciparum prevalence in two communities of Muheza district North-eastern Tanzania: correlation between parasite prevalence, malaria interventions and rainfall in the context of re-emergence of malaria after two decades of progressively declining transmission. *Malar. J.* **17**, 1–10 (2018).
45. Bernard, J. *et al.* Equity and coverage of insecticide-treated bed nets in an area of intense transmission of Plasmodium falciparum in Tanzania. *Malar. J.* **8**, (2009).
46. Mappin, B. *et al.* Standardizing Plasmodium falciparum infection prevalence measured via microscopy versus rapid diagnostic test. *Malar. J.* **14**, 460 (2015).
