# Supplementary material for: Severe outcomes of malaria in children under time-varying exposure
Source: Nat Commun. 2024 May 14;15:4069. doi: 10.1038/s41467-024-48191-7 (PMC11094066; doi:10.1038/s41467-024-48191-7)
Supplement: Supplementary file 3 — Reporting Summary [file 41467_2024_48191_MOESM3_ESM.pdf]

nature portfolio

Corresponding author(s): Pablo M De Salazar

Last updated by author(s): April 18th 24

Reporting Summary

Nature Portfolio wishes to improve the reproducibility of the work that we publish. This form provides structure for consistency and transparency in reporting. For further information on Nature Portfolio policies, see our Editorial Policies and the Editorial Policy Checklist.

Statistics

For all statistical analyses, confirm that the following items are present in the figure legend, table legend, main text, or Methods section.

n/a	Confirmed	
☐	☒	The exact sample size (*n*) for each experimental group/condition, given as a discrete number and unit of measurement
☐	☒	A statement on whether measurements were taken from distinct samples or whether the same sample was measured repeatedly
☐	☒	The statistical test(s) used AND whether they are one- or two-sided<br>*Only common tests should be described solely by name; describe more complex techniques in the Methods section.*
☐	☒	A description of all covariates tested
☐	☒	A description of any assumptions or corrections, such as tests of normality and adjustment for multiple comparisons
☐	☒	A full description of the statistical parameters including central tendency (e.g. means) or other basic estimates (e.g. regression coefficient) AND variation (e.g. standard deviation) or associated estimates of uncertainty (e.g. confidence intervals)
☐	☒	For null hypothesis testing, the test statistic (e.g. *F*, *t*, *r*) with confidence intervals, effect sizes, degrees of freedom and *P* value noted<br>*Give P values as exact values whenever suitable.*
☒	☐	For Bayesian analysis, information on the choice of priors and Markov chain Monte Carlo settings
☐	☒	For hierarchical and complex designs, identification of the appropriate level for tests and full reporting of outcomes
☒	☐	Estimates of effect sizes (e.g. Cohen's *d*, Pearson's *r*), indicating how they were calculated

Our web collection on statistics for biologists contains articles on many of the points above.

Software and code

Policy information about availability of computer code

Data collection	NA
Data analysis	Rstudio

For manuscripts utilizing custom algorithms or software that are central to the research but not yet described in published literature, software must be made available to editors and reviewers. We strongly encourage code deposition in a community repository (e.g. GitHub). See the Nature Portfolio guidelines for submitting code & software for further information.

Data

Policy information about availability of data

All manuscripts must include a data availability statement. This statement should provide the following information, where applicable:

- Accession codes, unique identifiers, or web links for publicly available datasets
- A description of any restrictions on data availability
- For clinical datasets or third party data, please ensure that the statement adheres to our policy

Model code, plotting code, and simulation data are available at https://github.com/PDeSalazarSwissTPH/SevereMalaria. Empirical data used in this analysis have been curated and uploaded to the Harvard Dataverse. Correspondence and requests for materials should be addressed to the KEMRI Wellcome Data Governance Committee (dgc@kemri-wellcome.org). These data are available through a formal requesting process to the KEMRI Institutional Data Access/Ethics Committee. Guideline details can be found on the KEMRI Wellcome website

Research involving human participants, their data, or biological material

Policy information about studies with human participants or human data. See also policy information about sex, gender (identity/presentation), and sexual orientation and race, ethnicity and racism.

Reporting on sex and gender	Data on hospital admission included sex (reported by a physician upon physical examination) but the variable is not used in the analyses. To date, there is no reports/evidence on sex influencing severe malaria.
Reporting on race, ethnicity, or other socially relevant groupings	Data used in the analysis of the manuscript does not include reporting on race or ethnicity.<br>Analyses were performed disaggregating by place of residence in the three countries, Tanzania, Uganda and Kenya.
Population characteristics	A total of 6506 malaria admissions in children aged 3 months to 9 years were used to define severe malaria<br>Data on admissions were grouped by year of age.
Recruitment	Human data on contemporary hospital admissions was obtained by reviewing hospital records retrospectively. No Informed consent was thus obtained from participants/tutors.<br>Data on prevalence surveys was obtained from previously published manuscripts
Ethics oversight	Kenya hospital surveillance: Kenya Medical Research Institute/ Scientific and Ethics Review Unit (KEMRI SERU) IRB numbers 1433, 3057, 3149; 1801, 2558, 2465, 3459 and 3771; Uganda hospital surveillance approved by the CDC as public health surveillance/non-research (NCEZID 031416), Tanzania hospital surveillance approved by the ethics committees of the National Institute for Medical Research, Tanzania, and the London School of Hygiene and Tropical Medicine. Kenya community parasite surveys KEMRI SERU IRB numbers 3149, 3592, 1801, 2558, 2675, 2801, and 3822; Uganda community prevalence surveys Vector Control Division Research Ethics Committee, MoH, Kampala: VCDREC/117.

Note that full information on the approval of the study protocol must also be provided in the manuscript.

Field-specific reporting

Please select the one below that is the best fit for your research. If you are not sure, read the appropriate sections before making your selection.

☒ Life sciences ☐ Behavioural & social sciences ☐ Ecological, evolutionary & environmental sciences

For a reference copy of the document with all sections, see nature.com/documents/nr-reporting-summary-flat.pdf

Life sciences study design

All studies must disclose on these points even when the disclosure is negative.

Sample size	Available record on paediatric malaria hospitalizations in 35-time sites. 6506cases
Data exclusions	No data was excluded
Replication	Simulated data was obtained after clusters of 1000-10000 simulations, repeated over >100 times. Data was eproducible
Randomization	Not relevant (we used only observational data fo description)
Blinding	Not relevant

Reporting for specific materials, systems and methods

We require information from authors about some types of materials, experimental systems and methods used in many studies. Here, indicate whether each material, system or method listed is relevant to your study. If you are not sure if a list item applies to your research, read the appropriate section before selecting a response.

Materials & experimental systems

n/a	Involved in the study
☒	☐ Antibodies
☒	☐ Eukaryotic cell lines
☒	☐ Palaeontology and archaeology
☒	☐ Animals and other organisms
☒	☐ Clinical data
☒	☐ Dual use research of concern
☒	☐ Plants

Methods

n/a	Involved in the study
☒	☐ ChIP-seq
☒	☐ Flow cytometry
☒	☐ MRI-based neuroimaging
